# Attitudes in music practice: a survey exploring the self-regulated learning processes of advanced Brazilian and Portuguese musicians

**DOI:** 10.3389/fpsyg.2024.1324100

**Published:** 2024-01-31

**Authors:** Camilla dos Santos Silva, Marcos Vinícius Araújo, Helena Marinho

**Affiliations:** ^1^Music Department, Universidade Estadual de Campinas (UNICAMP), Campinas, Brazil; ^2^Department of Communication and Art, University of Aveiro, Aveiro, Portugal; ^3^Institute of Ethnomusicology – Center for Studies in Music and Dance (Inet-MD), Aveiro, Portugal; ^4^Departamento de Música, Programa de Pós-graduação em Música, Universidade Federal do Rio Grande do Sul, Porto Alegre, Brazil

**Keywords:** self-regulated learning, music practice, survey, advanced musicians, exploratory factor analysis

## Abstract

**Introduction:**

This study aimed to investigate the Self-Regulated Learning behaviors of advanced Brazilian and Portuguese musicians and how these processes vary in terms of gender, nationality, musical instrument, quantity of practice, expertise, and professional experience.

**Methods:**

300 participants fully completed the 22-item questionnaire “Attitudes in music practice”. The sample comprised of 54.3% males, 44.0% females, and 1% non-binary; 0.7% did not respond. 68.0% (*n* = 204) were Brazilian, and 32.0% (*n* = 96) were Portuguese. The mean age was 32.70 years old (SD = 11.261), the mode was 22 years old, with a range of 18 to 66 years. Data analysis procedure included exploratory factor analysis, internal consistency, independent sample t test, analysis of variance (ANOVA), and chi-square tests.

**Results:**

Exploratory Factor Analysis generated three factors: Practice Organization, Personal Resources, and External Resources. The results report there are no differences in SRL scores in terms of gender, nationality, and musical instrument. However, One-way ANOVA test results convey differences in SRL scores and the quantity of practice and expertise with those musicians who reported practicing for longer periods scoring more highly than participants who declared spending less time on daily practice.

**Discussion:**

The results for the expertise variables suggest that more experienced and older musicians scored higher in Personal Resources and lower in External Resources indicating that, as musicians gain in experience, their metacognitive processes become more evident than the social factors of their performance.

## 1 Introduction

Musicians who achieve high levels of proficiency invest a significant amount of their time practicing their instrument and continue to train in order to maintain excellent performance levels. However, the quality of this practice is of utmost importance as the outcomes do not solely depend on the quantity of hours invested (Ericsson et al., 1993; Williamon and Valentine, 2000; Byo and Cassidy, 2008; Bonneville-Roussy and Bouffard, 2015). Research into musical practice has focused on investigating the factors rendering practice efficiency beyond quantity of practice, discussing processes such as how to set personal goals, sustain focus, and persevere in error correction as well as how best to address challenging musical sections (Ericsson et al., 1993; Araújo, 2016; How et al., 2022).

A comprehensive review of music practice research from 1928 to 2020, conducted by How et al. (2022), demonstrates the influence of psychological methodologies on this domain. Their analysis reveals that popular topics and extensively cited articles revolve around psychological constructs such as deliberate practice, motivation, and self-regulation. The role of self-regulation, defined by Zimmerman (2000), p. 14 as “self-generated thoughts, feelings, and actions that are planned and cyclically adapted to the attainment of personal goals” in learning processes, may be temporally distributed as described in Zimmerman’s cyclical model (Zimmerman, 2000; Zimmerman and Campillo, 2003). Zimmerman’s model comprises three phases: Forethought, Performance/Volitional Control, and Reflection. In the Forethought phase, learners employ various actions and strategies for task analysis and goal setting. This is motivated by their self-efficacy beliefs, outcome expectations, and perceived value of the task. Self-control and self-observation are essential during the Performance/Volitional Control phase for the proper employment of learning strategies. This includes focus maintenance, self-monitoring, and other metacognitive subprocesses. During the third phase, Self-Reflection, the learner conducts self-evaluation and manages self-reactions. These self-reflective conclusions feed into the next learning cycle, influencing the subsequent Forethought phase of the model (Zimmerman, 2000).

The model was adapted to music learning and performance by McPherson and Zimmerman (2002, 2011) and extensively applied to music research in order to investigate metacognitive aspects of music practice and performance (How et al., 2022). Even advanced musicians can benefit from SRL strategies and improve their musical performance and daily practice (Clark and Williamon, 2011; Pike, 2017; López-Íñiguez and McPherson, 2020).

From a cognitive point of view, the most conventional definition of a musician is someone who has the ability to play an instrument or sing, this ability being acquired through years of practice (Hallam, 2010; Zhang et al., 2020). Thus, most studies consider the amount of deliberate practice, which is formal and supervised by a teacher, as a primary factor in the development of musical expertise. In Western music, this type of practice occurs mainly in conservatories and higher education music institutions.

According to previous literature (Ericsson et al., 1993), advanced musicians were defined as those who had at least 10 years of experience with their main instrument. However, as research in musical practice has increased, musicians enrolled in higher education institutions, such as universities, professional conservatories and other tertiary institutions, who are preparing to become professional musicians or already working professionally, have been also designated as advanced musicians (Papageorgi et al., 2010; Araújo, 2016). In this study, we will use “advanced musicians” to refer to these individuals. Advanced musicians exhibit practice skills similar to deliberate practice, engage in a focused manner during practice, and have received adequate training to organize their practice based on the technical, theoretical and interpretative aspects of music (Williamon and Valentine, 2000; Miksza, 2015). These musicians have also been described as *elite musicians* (De Bruin, 2019; Kegelaers and Oudejans, 2020; Mornell et al., 2020), *expert musicians* (De Bruin, 2017; Fasano et al., 2020), *undergraduate musicians* (Clark and Williamon, 2011; Zhukov, 2012; McPherson et al., 2019), *professional musicians* (Dos Santos and Gerling, 2011; Pike, 2017), and *college musicians* (Kim, 2010; Boucher et al., 2020, 2021).

Regarding the development of musical expertise, it is possible to consider it in relationship with the training period in higher music education. Before entering the higher education course, there is a specific test to assess the minimum musical skills required to enter the course, skills usually developed through years of practice in music schools and conservatories prior to university. Placement in the professional job market usually occurs after completing an undergraduate course in musical performance (Creech et al., 2008).

Developing musical skills is a comprehensive journey involving a combination of theoretical and practical experiences, with relevant aspects such as a first solid foundation in the theoretical and practical foundations of music, including the study of music theory, sheet music reading, counterpoint, harmony, musical analysis, history of music and constant auditory training through music perception classes. During this time, students also have constant instrument lessons and opportunities to play solo and in an ensemble, a period that culminates with the first public audition. Up to this point, we consider naming the category as students, being those who have not yet performed the mid-course recital. The mid-course recital already has a public character and is normally the first public performance of repertoire prepared under the guidance of a specialist teacher.

After the mid-course recital, musicians can be considered pre-professionals because they are already in the final year of their undergraduate course, in preparation for the final recital. Following the rationale, individuals who finished their undergraduate music course were considered professionals, as they were, by definition, ready for the job market. This categorization is justified as a way of expanding the current notion of musical expertise development, and it is the one we used in the present study.

The literature has extensively studied the quantitative measuring of SRL behaviors in advanced musicians, especially in the last decade (Ritchie and Williamon, 2013; Bonneville-Roussy and Bouffard, 2015; Miksza and Tan, 2015; Araújo, 2016; Ersozlu et al., 2017; Hatfield et al., 2017; Volioti and Williamon, 2017; Topoğlu and Topoğlu, 2018; Boon, 2020; Peistaraite and Clark, 2020; Nusseck and Spahn, 2021; Liu, 2023a). A recent review on musical practice by How et al. (2022) reports that 66.2% of the retrieved articles applied quantitative methods, with questionnaires emerging as the most common instrument type (47.4%). Initially, descriptive-correlational studies in this field employed instruments developed for general education or adaptations of these instruments, such as the Pintrich and de Groot Motivated Strategies for Learning Questionnaire (MSLQ) (Pintrich and De Groot, 1990), applied by McPherson and McCormick (1999, 2000), and Nielsen (2004, 2012) or the Self-Regulated Learning Interview Schedule (Zimmerman and Martinez-Pons, 1986), used by Clark and Williamon (2011) and Ritchie and Williamon (2013). However, music practice also involves specific behaviors and task-related demands, which prompted the need to develop a measurement instrument tailored to SRL processes related to musical learning (Miksza, 2012; Araújo, 2016).

The Self-Regulated Practice Behavior scale was designed and validated (α = 0.76 to 0.90) for measuring the self-regulatory behaviors of beginner and intermediate musicians (Miksza, 2012). This scale has since been translated and validated in Turkish (Ersozlu and Miksza, 2015; *α* =0.62 to 0.90), Portuguese (Madeira et al., 2018; *α* = 0.71 to 0.84), and Chinese (Zhang et al., 2023; *α* = 0.77 to 0.86). Subsequently, Araújo (2016) designed the “Attitudes and Sensations in Music Practice” questionnaire for advanced musicians to assess SRL behaviors and Flow sensations in advanced musicians, which has since been validated and applied in both Portuguese and English (*α* = 0.86). However, considering the best practices based on recent evidence (Costello and Osborne, 2005; Worthington and Whittaker, 2006; Howard, 2016; Rogers, 2022), we deem it necessary to replicate Araújo’s study, updating the validation procedures of the “Attitudes in Music Practice” scale (Araújo, 2016) as regards Exploratory Factorial Analysis, before conducting Confirmatory Factor Analysis (CFA), to avoid model specification errors. These updates will be described in section 2.4.

Quantitative studies have investigated if there are differences or relationships between SRL processes and variables like quantity of practice (Ritchie and Williamon, 2013; Bonneville-Roussy and Bouffard, 2015; Araújo, 2016; Topoğlu and Topoğlu, 2018; Boon, 2020; Zhang et al., 2023), gender (Topoğlu and Topoğlu, 2018; Nusseck and Spahn, 2021; Liu, 2023a), musical instrument (Nielsen, 2004; Liu, 2023a), expertise (Boon, 2020; Kaleli, 2021), and age (Bonneville-Roussy and Bouffard, 2015). We did not find evidence of correlations or differences between SRL scores and nationality in the reviewed quantitative studies, besides Araújo’s (2016).

Thus, the purpose of the study is to explore the SRL behaviors of advanced musicians from Brazil and Portugal and how these processes vary according to gender, musical instrument, quantity of practice (measured by hours of practice per day and days of practice per week), expertise (determined from information about the participants’ formal music education), professional experience (measured by years since first public music performance), age, and nationality.

In light of previous findings in the SRL literature, we formulated the following four hypotheses. First, Exploratory Factorial Analysis will identify the same dimensions as the first study by Araújo (2016); second, similar to the first application of the questionnaire (Araújo, 2016), the SRL scores will report no significant differences regarding gender, nationality, and musical instrument; third, advanced musicians who declare spending more hours per day in music practice will score higher in SRL processes; fourth, more experienced participants will score higher in SRL processes.

## 2 Materials and methods

### 2.1 Instrument

The Attitudes in Music Practice questionnaire (Araújo, 2016) applied in this study comprises 22 items (see Supplementary material) and was validated both in Portuguese and English. These items were designed to assess various aspects of self-regulated practice behaviors, such as the management and evaluation of practice goals (e.g., ‘I set specific goals for my practice sessions’), time management and physical environment structuring (e.g., ‘I plan the time of my practice sessions’), strategy selection (e.g., ‘I am aware of the strategies that I use during practice’), self-efficacy beliefs (e.g., ‘I am able to achieve my practice goals satisfactorily’), external causal attributions (e.g., ‘I cannot reach my practice goals without the support of some external factors - peers, teachers, materials, environment’), help-seeking (e.g., ‘I request help from others [teachers, peers, composers, musicologists and specialists]’) and external resources (e.g., ‘I seek information from several sources - books, CDs, videos, internet, biographies, arts, etc. to support my study’).

Participants were required to rate their self-regulated practice behaviors on a 5-point Likert-type scale, based on the frequency of behaviors, ranging from 1-never to 5-always in some items, and level of agreement ranging from 1-completely disagree to 5-completely agree in other items (see Appendix for the complete questionnaire). The questionnaire also included a demographic data section, which requested information about the participants’ age, gender, nationality, formal education, musical instrument, practice time (hours of practice per day and days of practice per week), and how many years from their first public musical performance.

### 2.2 Data retrieval

The questionnaire and the Formal Consent form (approved by the local Ethics Committee) were hosted by the Lime Survey platform, managed by the University of Aveiro, and with completed responses submitted through a hyperlink. Prior to its dissemination, we asked 9 SRL researchers and advanced musicians to answer the questionnaire between January 8 and January 9, 2021 to identify eventual difficulties in understanding experienced by participants. The responses returned by the pilot study were removed from the platform to protect the final data.

Data collection occurred during the periods of lockdown in Portugal and Brazil and all contact with participants was therefore virtual. The researchers prepared two invitations to participate in this research project, one addressed to teachers from higher education institutions, distributed by email, and the other for musicians in general and for students attending these institutions, which was disseminated by e-mail and social media in order to attract a wide range of participants.

The questionnaire link was active from January 10, 2021 until May 7, 2021. 476 people answered the questionnaire; only 306 answered the complete questionnaire correctly.

### 2.3 Sample

The inclusion criteria consisted of Brazilian and Portuguese advanced musicians. Based on this criteria, we removed six participants because they were under 18 years old and had limited musical instrument experience. The final sample consisted of 300 participants (54.3% male, 44.0% female, 1% non-binary, and 0.7% did not respond). 68.0% (*n* = 204) of the participants were Brazilian, and 32.0% (*n* = 96) were Portuguese.

Their ages ranged from 18 to 66 years old, with a mean of 32.70 years old (SD = 11.261), mode 22 years old; with most participants in the age range between 26 and 35 years of age (*n* = 104, more in Table 1).

**Table 1:** Age range.

Age range	Frequency	Percentage
18–25 years old	96	32.0%
26–35 years old	104	34.7%
36 +	99	33.3%

Since the educational system in Brazil differs from Portugal, we organized this information from participants into categories of expertise: participants in their first 2 years of professional music education (undergraduate studies) were allocated to the Student category. Participants in their final year of undergraduate studies were classified as Pre-professionals, and participants who had completed undergraduate studies and/or undertook graduate studies were allocated to the Professional category.

The descriptive results identify how 54% of participants fell into the Professional category. Table 2 presents the results for this variable.

**Table 2:** Expertise categories.

Expertise	Frequency	Percentage
Student	58	19.3
Pre-Professional	80	26.7
Professional	162	54.0

Regarding years of experience since their first public concert, the majority of participants (76.0%) declared having 10 or more years of professional performance experience and with the majority beginning their instrument lessons when they were 12 years old or younger (56.7%).

As regards musical instruments, 49.7% of participants declared playing plucked string instruments (*n* = 149), 15.3% wind instruments (*n* = 46), 12.7% bowed strings (*n* = 38), 10.7% keyboards (*n* = 32), 8% voice (*n* = 24), 1.7% percussion (*n* = 5) and 1.7% conductors (*n* = 5). Due to analysis requirements regarding the minimum number of participants in each category (Field, 2018), percussionists and conductors were not included in the inferential analysis. One participant did not register information about the instrument (Table 3).

**Table 3:** Musical instrument categories.

Instrument	Frequency	Percentage
Plucked strings	149	49.8
Keyboards	32	10.7
Bowed strings	38	12.7
Voice	24	8.0
Wind	46	15.4
Percussion	5	1.7
Conductors	5	1.7
Missing	1	0.3

When asked about the practice quantity, participants answered how many days per week they practiced, and how much time per day (in hours). 13.3% declared practicing 1 to 2 days per week, 23.2% practiced 3 to 4 times per week, 37.3% of participants (*n* = 112) declared practicing 5 to 6 days a week, and 26.0% (*n* = 78) practiced daily. The majority of participants declared practicing between 1 and 2 h per day (27.7%). Table 4 reports the length of daily practice hours.

**Table 4:** Practice hours per day.

Practice time per day	Frequency	Percentage
<1 h	48	16.0
1–2 h	83	27.7
4–3 h	80	26.7
3–4 h	59	19.7
> 4 h	30	10.0

### 2.4 Data analysis procedure

#### 2.4.1 Exploratory factor analysis

Exploratory Factor Analysis (EFA) served to explore the structure of the scale and assess its internal reliability. The dispersion matrix was generated by polychoric correlations (Muthén and Kaplan, 1985, 1992; Baglin, 2014). Researchers suggest employing polychoric correlations when conducting EFA on data derived from ordinal variables (Baglin, 2014). We assessed sampling adequacy and factorability according to the Kaiser-Meyer-Olkin (KMO) index and significance by Bartlett’s test of sphericity (Rogers, 2022). The extraction method adopted was Robust Diagonally Weighted Least Squares (RDWLS–Asparouhov and Muthen, 2010). This estimator is most suitable for categorical data and is robust in handling deviations from normality (DiStefano and Morgan, 2014). To determine the appropriate number of factors, we deployed parallel analysis with random permutation of observed data (Timmerman and Lorenzo-Seva, 2011; Baglin, 2014) that has proven to be more effective than traditional methods in accurately determining the actual number of dimensions (Timmerman and Lorenzo-Seva, 2011; Baglin, 2014). Items with factor loadings ≥0.30 were considered relevant and included in the model. We implemented the Robust Promin rotation method (Lorenzo-Seva and Ferrando, 2019). It is advisable to select oblique rotation methods for multidimensional scales, as most factors within these scales tend to have some level of interrelation, and orthogonal rotations presume that the factors are independent (Fabrigar et al., 1999; Fabrigar and Weneger, 2011; Baglin, 2014; Lloret-Segura et al., 2014; Howard, 2016). Model adequacy was assessed by the Root Mean Square Error of Approximation (RMSEA), Comparative Fit Index (CFI), and Non-Normed Fit Index - Tucker-Lewis Index (NNFI). According to the literature (Fabrigar et al., 1999; Brown, 2015), the RMSEA values should be less than 0.08, and the CFI and NNFI results should be above 0.90. EFA was performed using the software FACTOR version 12 (Lorenzo-Seva and Ferrando, 2006; Ferrando and Lorenzo-Seva, 2017; Rogers, 2022).

#### 2.4.2 Internal consistency

We tested the internal consistency of each factor by McDonald’s Omega coefficients (Hayes and Coutts, 2020) and the Composite Reliability Index (Raykov, 1997; Valentini and Damásio, 2016; with ≥0.60 considered satisfactory). These calculations were performed by Jasp software (version 0.16.4) for McDonald’s Omega, and the Composite Reliability Calculator for the Composite Reliability Index. We did not apply Cronbach’s alpha coefficient due to current discussions questioning its suitability for the types of data and models deployed in psychological research, which often violate the assumptions made by this coefficient (McNeish, 2018; Hayes and Coutts, 2020). Especially in scales with a smaller number of items, Cronbach’s alpha may report reliability lower than the scale actually attains (Zinbarg et al., 2006; McNeish, 2018).

#### 2.4.3 Independent sample t test

An independent sample t-test was conducted to investigate how the “Attitudes in Music Practice” scale scores differed between groups, according to Gender and Nationality. The assumption of homogeneity of variance was evaluated using Levene’s test and, when violated, we applied Welch’s statistic. Bootstrapping procedures (1,000 re-samples; 95% IC BCa) were performed to correct for the non-normality of the sample distribution and to increase the reliability of the results (Tan and Tan, 2010). We calculated the effect size according to Hedges g to account for bias in unbalanced samples in keeping with Cohen’s benchmarks (Cohen, 1988); g = 0.2, 0.5, and 0.8 correspond to small, medium, and large effects (Lakens, 2013). This data analysis made recourse to IBM SPSS Statistics software (Version 28).

#### 2.4.4 Analysis of variance (ANOVA)

One-way ANOVA evaluated the potential differences in musical practice attitudes based on the scores of the three dimensions (Practice Organization, Personal Resources, External Resources) within group variables: instrument, practice hours per day, days of practice per week, number of concerts per year, expertise, and age. The assumption of variance homogeneity was assessed using Levene’s test, and post-hoc evaluation was performed using the Bonferroni correction for multiple comparisons (Field, 2018). We then applied bootstrapping procedures (1,000 re-samplings; 95% IC BCa) to obtain a higher level of result reliability, correcting any normality deviations from the sample distribution and returning more robust confidence intervals for the mean differences (Haukoos and Lewis, 2005; Tan and Tan, 2010). The coefficient ω^2^ represented effect size, according to Cohen’s benchmarks (Cohen, 1988, 0.01 = small; 0.06 = medium; 0.14 = large). We made recourse to IBM SPSS Statistics (Version 28) as the statistical software for this analysis.

#### 2.4.5 Chi-square

We assessed the relationships among the categorical variables by the chi-square test of association. These relationships were deemed significant when the adjusted residuals were > 1.96 (regardless of sign).

## 3 Results

### 3.1 Construct validity and internal consistency

#### 3.1.1 Exploratory factor analysis

We examined the dataset to identify any inconsistent and/or missing values related to participant responses to scale items with no such inconsistencies detected. However, we excluded from this analysis participants who did not respond to all the scale items. Therefore, for analyses related to the internal structure of the instrument, the total sample size was *n* = 297. Table 5 presents the univariate descriptive analysis for the 22 original items of the “Attitudes in Musical Practice” scale.

**Table 5:** EFA univariate descriptive analysis.

	Item	Mean	Confidence Interval-95%		Variance	Skewness	Kurtosis (Zero centered)
			Lower	Upper			
1	I set goals for my practice sessions	3.95	3.82	4.10	0.897	−0.628	−0.195
2	I set short term goals (minutes, hours, days)	3.70	3.53	3.87	1.240	−0.621	−0.342
3	I set long-term goals (weeks, months, years)	3.89	3.74	4.05	1.073	−0.732	−0.169
4	I set specific goals for my practice sessions	3.91	3.78	4.06	0.903	−0.665	−0.010
5	I understand that my goals are challenging	4.14	4.02	4.27	0.726	−0.780	0.094
6	I use specific strategies related to my practice goals	4.12	4.01	4.25	0.630	−0.841	0.959
7	I am aware of the strategies that I use during practice	4.23	4.12	4.35	0.591	−0.967	1.326
8	I use strategies that have been effective in the past	4.13	4.01	4.25	0.619	−0.694	0.336
9	I know when and in which contexts my strategies will be most effective	3.88	3.75	4.02	0.804	−0.581	0.053
10	I understand the nature and demands of my musical activities	4.38	4.27	4.50	0.614	−1.129	0.599
11	I know what I must do in order to complete my musical activities satisfactorily	4.02	3.89	4.16	0.794	−0.741	0.150
12	I plan the order of the activities of my practice sessions	3.64	3.47	3.81	1.293	−0.453	−0.702
13	I plan the time of my practice sessions	3.45	3.28	3.63	1.386	−0.324	−0.785
14	I organize the physical environment of my practice sessions	3.80	3.63	3.98	1.376	−0.746	−0.346
15	I evaluate the progress made toward my goals	3.73	3.58	3.88	1.003	−0.476	−0.408
16	I seek information from several sources (book, CDs, videos, internet, biographies, arts, etc.) to support my study	3.97	3.82	4.12	1.036	−0.734	−0.263
17	I request help from others (teachers, peers, composers, musicologists and specialists)	3.62	3.47	3.79	1.163	−0.167	−0.936
18	I am able to achieve my practice goals satisfactorily	3.80	3.68	3.92	0.637	−0.424	0.069
19	I cannot reach my practice goals without the support of some external factors (peers, teachers, materials, environment)	3.52	3.35	3.70	1.387	−0.472	−0.641
20	I understand my strengths and weaknesses	4.22	4.12	4.34	0.552	−0.687	0.055
21	I practice in order to improve my musical skills	4.50	4.40	4.61	0.499	−1.496	2.435
22	I practice in order to achieve high ratings (e.g., grades) and positive feedback	3.30	3.13	3.49	1.465	−0.228	−0.825

The first stage of analysis included all the 22 scale items. Bartlett’s sphericity test returned a significant result [*χ*^2^ = 3219.2 (df = 231; *p* = 0.000010)] and the KMO measure (0.85772) was also above that recommended (Howard, 2016). The robust goodness of fit statistics were satisfactory (RMSEA = 0.062; NNFI; Tucker and Lewis = 0.963; CFI = 0.970) with the parallel analysis indicating the extraction of two factors. According to the two-factor model, all items achieved a relevant factor loading (≥ 0.30); however, items 6 (“I use specific strategies related to my practice goals”) and 17 (“I request help from others teachers, peers, composers, musicologists and specialists”) reported cross loading (see Supplementary Table S1).

We carried out further analysis to undertake the extraction of the three dimensions based on Araújo’s preliminary study from 2016. The three-factor model maintained the Bartlett [*χ*^2^ = 3219.2 (df = 231; *p* = 0.000010)] and KMO (0.85772) results, and the robust goodness of fit indices returned improvements (RMSEA = 0.043; NNFI; Tucker and Lewis = 0.982; CFI = 0.987). Item 17 (“I request help from others - teachers, peers, composers, musicologists and specialists”) loaded above 0.30 on only one dimension. However, item 6 still presented cross loading (Supplementary Table S2).

The model also reflects theoretical inconsistency in the distribution of items across factors. Item 5 (“I understand that my goals are challenging”) was allocated to factor 3, which groups items related to the External Factors dimension. This distribution would thus hinder the discussion of this dimension as a whole. Therefore, items 5 and 6 (“I use specific strategies related to my practice goals”) were excluded and we performed a new EFA.

Both Bartlett’s test of sphericity and the KMO results were acceptable [*χ*^2^ = 2851.7 (df = 210; *p* = 0.000010); KMO = 0.85718]. The robust goodness of fit indices demonstrated a better fit than the previous models: (RMSEA = 0.037; NNFI; Tucker and Lewis = 0.986; CFI = 0.990). Table 6 displays the factorial loadings of this updated model.

**Table 6:** Factor loadings.

	Item	F1	F2	F3
1	I set goals for my practice sessions	0.969		
2	I set short term goals (minutes, hours, days)	0.736		
3	I set long-term goals (weeks, months, years)	0.385		
4	I set specific goals for my practice sessions	0.956		
12	I plan the order of the activities of my practice sessions	0.709		
13	I plan the time of my practice sessions	0.703		
14	I organize the physical environment of my practice sessions	0.451		
15	I evaluate the progress made toward my goals	0.636		
7	I am aware of the strategies that I use during practice		0.743	
8	I use strategies that have been effective in the past		0.484	
9	I know when and in which contexts my strategies will be most effective		0.836	
10	I understand the nature and demands of my musical activities		0.804	
11	I know what I must do in order to complete my musical activities satisfactorily		0.861	
18	I am able to achieve my practice goals satisfactorily		0.461	
20	I understand my strengths and weaknesses		0.679	
16	I seek information from several sources (books, CDs, videos, Internet, biographies, arts, etc.) to support my study			0.449
17	I request help from others (teachers, peers, composers, musicologists and specialists)			0.717
19	I cannot reach my practice goals without the support of some external factors (peers, teachers, materials, environment)			0.635
21	I practice in order to improve my musical skills			0.403
22	I practice in order to achieve high ratings (e.g., grades) and positive feedback			0.325

Thus, Factor 1 comprises the items related to Practice Organization processes that incorporate the behavioral determinants of SRL (Zimmerman, 1989). Factor 2, designated Personal Resources by Araújo (2016), gathers those processes associated with personal determinants, and Factor 3, External Resources, refers to influences from the surrounding environment, which is the third determinant described by Zimmerman (1989). This analysis returns extraction scores similar to those of Araújo’s (2016) primary study. Thus, analyses below will not contain items 5 and 6.

#### 3.1.2 Internal consistency

Factor 1 internal consistency resulted in McDonald’s *ω* = 0.90; [CI 95% (0.88–0.91)], composite reliability = 0.918; Factor 2 *ω* = 0.78 [CI 95% (0.75–0.88)], composite reliability = 0.841; and Factor 3 *ω* = 0.93 [CI 95% (0.93–0.94)], composite reliability = 0.953.

#### 3.1.3 Total Scores

Participants obtained a mean score of 30.13 (SD = 5.93) in Practice Organization, 28.69 (SD = 3.93) in Personal Resources, and 18.98 (SD = 3.22) in External Resources. Supplementary Table S17 contains this data and total scores.

### 3.2 Differences in the SRL scores deriving from the variables measured

#### 3.2.1 Gender

The T test results for gender return no significant differences in the SRL scores between groups across any of the dimensions [Practice Organization: *t* (291.802) = −1.509 *p* = 0.132 IC 95% Bca: −2.36; 0.343; Personal Resources: *t* (292) = 1.069, *p* = 0.286 (IC 95% Bca: −0.401; 1.424); External Resources: *t* (291.366) = −0.937, *p* = 0.350 (IC 95% Bca: −1.023; 0.345)]. These descriptive statistics are made available in Supplementary Table S3. Considering the low sample size of participants in the non-binary category, we were compelled to conduct the test only with the male and female categories.

#### 3.2.2 Nationality

The SRL scores when organized by nationality also presented no significant differences according to the T test results [Practice Organization: *t* (295) = −0.155 *p* = 0.877 (IC 95% Bca: −1.505; 1.251), Personal Resources: *t* (295) = −1.257, *p* = 0.210 (IC 95% Bca: −1.550; 0.302); External Resources: *t* (223.718) = 0.818, *p* = 0.415(IC 95% Bca: −0.475; 1.021)]. In turn, Supplementary Table S4 sets out these descriptive statistics.

#### 3.2.3 Musical instrument

Levene’s tests describe a homogeneity of variance across every dimension: Practice Organization [Levene (4, 284) = 0.253, *p* = 0.908], Personal Resources [Levene (4, 283) = 0.624, *p* = 646], External Resources [Levene (4, 284) = 1.744, *p* = 140].

The ANOVA results for differences between musical instruments and SRL were also statistically non-significant [Practice Organization: *F* (4, 284) = 0.469, *p* = 0.758; Personal Resources: *F* (4, 283) = 1.24, *p* = 0.290; External Resources: *F* (4, 284) = 1.10, *p* = 0.354]. These descriptive statistics are available in Supplementary Table S5.

#### 3.2.4 Quantity of practice: practice hours per day

Levene’s tests report a homogeneity of variance across all dimensions: Practice Organization [Levene, (4, 294) = 0.795, *p* = 0.529]; Personal Resources [Levene, (4, 292) = 1.053, *p* = 0.380]; External Resources [Levene, (4, 294) = 1.554, *p* = 0.187]. The descriptive statistics for all groups and dimensions are set out in Supplementary Table S6 in the Appendix.

The ANOVA results demonstrate significant differences with small size effects between the groups in Practice Organization [*F* (4, 294) = 3.818, *p* = 0.005, ω^2^ = 0.04]. Specifically, there are differences in the mean scores between musicians who practice less than 1 h per day (*M* = 28.55, SD = 6.05) and those who practice more than 4 h per day (*M* = 32.77, SD = 5.69) as well as between participants practicing between 1 and 2 h per day (*M* = 29.17, SD = 6.26) and those practicing more than 4 h per day (M = 32.77, SD = 5.69).

Regarding the External Resources dimension, there are medium size effect differences (F(4, 294) = 7.608, *p* < 0.001, ω^2^ = 0.08). These differences emerge when comparing musicians who practice less than 1 h per day (*M* = 17.09, SD = 3.49) with those who practice within the range of 2 to 3 h (*M* = 19.44, SD = 3.34) and between 3 and 4 h (*M* = 20.14, SD = 2.70), as well as with participants practicing over 4 h per day (*M* = 19.60, SD = 2.52). Additionally, the results also detail differences between participants practicing between 1 and 2 h per day (*M* = 18.55, SD = 3.03) and those practicing for between 3 and 4 h per day (*M* = 20.14, SD = 2.70). Supplementary Table S7 describes the post-hoc tests deploying Bonferroni correction and CI through Bootstrapping.

#### 3.2.5 Quantity of practice: practice days per week

All dimensions display homogeneity of variance: Practice Organization [Levene, (3, 295) = 0.324, *p* = 0.808]; Personal Resources [Levene, (3, 293) = 2.465, *p* = 0.062]; External Resources [Levene, (3, 295) = 0.254, *p* = 0.859] with the complete descriptive statistics set out in Supplementary Table S8 (see Appendix).

The ANOVA results point to small effect differences in the Practice Organization dimension [*F*(3, 295) = 6.157, *p* < 0.001, ω^2^ = 0.049]. In particular, there are significant differences between the categories of musicians who practice between 1 and 2 days per week (*M* = 26.88, SD = 6.51) and musicians who practice between 5 and 6 days per week (*M* = 30.83, SD = 5.74) as well as musicians practicing daily (*M* = 31.35, SD = 5.39).

In the External Resources dimension [*F* (3, 295) = 9.810, *p* < 0.001, ω2 = 0.081], there are significant medium size differences between musicians practicing between 1 and 2 days per week (*M* = 16.95, SD = 3.08) and musicians practicing between 5 and 6 days per week (*M* = 19.28, SD = 3.04) as well as musicians practicing daily (*M* = 20.05, SD = 3.37). Differences also appear between musicians practicing between 3 and 4 days per week (*M* = 18.47, SD = 2.82) and their peers practicing daily (*M* = 20.05, SD = 3.37). These post-hoc tests appear in Supplementary Table S9.

#### 3.2.6 Expertise

After excluding the possibility of non-homogeneity of variance across all dimensions [Practice Organization: Levene (2, 296) = 0.412, *p* = 0.663; Personal Resources: Levene (2, 294) = 0.186, *p* = 0.831; External Resources: Levene (2, 296) = 1.225 *p* = 0.295], we conducted a one-way ANOVA test to examine the differences between levels of expertise. The corresponding descriptive results are available in Supplementary Table S10.

The ANOVA results reveal significant differences with a small size effect in the Personal Resources Dimension [*F*(2, 294) = 5.659, *p* = 0.004; ω^2^ = 0.030] between the Pre-Professionals (*M* = 27.65; SD = 3.62) and the Professionals (*M* = 29.36; SD = 3.87). In terms of the External Resources dimension, the results convey significant differences with small size effects [*F*(2, 296) = 8.085, *p* < 0.001; ω^2^ = 0.045] between Students (*M* = 20.21; SD = 3.31) and Professionals (*M* = 18.35; SD = 2.98). Supplementary Table S11 (in the Appendix) details the post-hoc tests for this variable.

#### 3.2.7 Age

Levene’s test confirms the homogeneity of variance across every dimension [Practice Organization: Levene (2, 296) = 2.475, *p* = 0.086; Personal Resources: Levene (2, 294) = 1.319, *p* = 0.269; External Resources: Levene (2, 296) = 0.232 *p* = 0.793] with the descriptive results provided in Supplementary Table S12 (Appendix).

The ANOVA results report small size differences in the Practice Organization dimension [F(2, 296) = 3.697; *p* = 0.26; ω^2^ = 0.018] between participants aged 26 to 35 years (*M* = 29.03, SD = 6.48) and those aged 36 years or older (*M* = 31.27, SD = 5.84). The Personal Resources dimension returned differences with a medium size effect [*F* (2, 294) = 12.333, p < 0.001; ω^2^ = 0.071] between musicians aged 18 to 25 years (*M* = 27.88, SD = 4.00) and those aged 36 years or older (*M* = 30.24, SD = 3.20), as well as between participants aged 26 to 35 years (*M* = 27.95, SD = 4.09) and those aged 36 years or older (*M* = 30.24, SD = 3.20). The External Resources dimension displays differences with a medium size effect between participants [*F* (2, 296) = 12.690, p < 0.001; ω^2^ = 0.073] aged 18 to 25 years (*M* = 20.28, SD = 2.89) and those aged 36 years or older (*M* = 18.21, SD = 3.12). Supplementary Table S13 presents the post-hoc tests.

#### 3.2.8 Time since the first public performance

Levene’s test results convey a homogeneity of variance in each of the three factors [Practice Organization: Levene (2, 296) = 0.030, *p* = 0.970; Personal Resources: Levene (2, 294) = 1.618, *p* = 0.200; External Resources: Levene (2, 296) = 0.319, *p* = 0.727]. These descriptive results are available in Supplementary Table S14 (Appendix).

The ANOVA results display medium sized differences in the Personal Resources dimension [*F* (2, 294) = 10.412, *p* < 0.001; ω^2^ = 0.060] between the “1 to 9 years” group (*M* = 26.75; SD = 4.47) and the “10 to 29 years” group (*M* = 28.74; SD = 3.78); between the “1 to 9 years” group and the “30 years or more” group (*M* = 30.18; SD = 3.29); and between the “10 to 29 years” group and the “30 years or more” group. In the External Resources dimension, we may report medium sized differences [*F* (2, 296) = 10.547, *p* < 0.001; ω^2^ = 0.060] between the “1 to 9 years” category (*M* = 20.73; SD = 2.88) and the “10 to 29 years” category (*M* = 18.83; SD = 3.15); and between the “1 to 9 years” category and the “30 years or more” category (*M* = 17.98; SD = 3.23). The complete post-hoc tests may be found in Supplementary Table S15 (Appendix).

### 3.3 Relationships between variables

#### 3.3.1 Practice hours per day X expertise

We then carried out a chi-square test of independence (3×5) to investigate the relationship between hours of study and participant expertise levels (student, pre-professional, and professional). The results identify a significant association between practice hours and expertise [*χ*^2^(8) = 16.812, *p* = 0.032, Cramer’s V = 0.16]. Analyses of the adjusted standardized residuals demonstrate that the student category statistically associated with more hours of study (1 to 2 h and 3 to 4 h of study per day). On the other hand, professionals were statistically associated with fewer hours of study (up to 1 h of study per day). Pre-professional participants did not show a significant relationship with any of the practice time categories. Supplementary Table S16 presents these estimates (see Appendix).

## 4 Discussion

This study set out to investigate the differences in the SRL scores achieved by advanced musicians according to age, gender, nationality, musical instrument, quantity of practice, expertise and quantity of professional experience. As described above, 300 participants completed a survey with the resulting data analyzed through parametric statistical tests. The findings report no statistically significant differences among the categories of gender, nationality and musical instrument. These results are consistent with those obtained in the first application of the same instrument (Araújo, 2016). Other studies of a similar design and sample, produced by Bonneville-Roussy and Bouffard (2015), Liu (2023a), Nusseck and Spahn (2021), and Topoğlu and Topoğlu (2018), also failed to encounter any gender differences.

Similar to our study, Nielsen (2004) reports no significant differences between instrument categories. In Liu’s (2023a) survey, brass players reported employing more learning strategies than keyboard players. In our study, woodwind and brass players were included in the same category due to the small sample size. Our results may not point out any significant differences existing as almost half of our sample were plucked string players.

The quantity of practice was measured according to two variables: the number of practice hours per day, and the practice days per week. Regarding the Practice Organization dimension, musicians that declared practicing more hours per day and more days per week obtained higher scores. In the Personal Resources dimension, there are no differences between the SRL scores and the categories of quantity of practice similar to the first application of this scale by Araújo (2016). However, the results for the External Resources dimension did contrast: while musicians who practiced for more than 4 h a day registered lower scores in this dimension (in comparison to participants who practiced between 3 and 4 h), and those who practiced every day of the week reported increased scores (in comparison with participants who practiced less days per week).

The literature has explored the correlation between the quantity of practice time and the self-regulatory behaviors exhibited by advanced musicians. Previous descriptive-correlational studies by Miksza and Tan (2015), Topoğlu and Topoğlu (2018), and Ritchie and Williamon (2013), as well as observational studies by Boon (2020) and the microanalysis study by Miksza et al. (2018), which measured the quantity of practice hours per day, also found that a greater number of practice hours corresponds to higher SRL scores.

However, when relating the quantity of practice hours per day to the expertise variable, the chi-square test of independence (3×5) here demonstrates that students were associated with more hours of practice per day than professionals. Furthermore, professionals were statistically associated with fewer hours of practice (up to 1 h of practice per day). Pre-professional participants did not show a significant relationship with any of the practice time categories.

These results are similar to those of Bonneville-Roussy and Bouffard (2015), Araújo (2016), and Dos Santos and Gerling (2011), suggesting that, as musicians gain more experience, their practice becomes more self-regulated, thereby reducing the practice time necessary to achieving goals. While musicians who practice for extended periods report greater recourse to SRL processes, more experienced musicians claim to practice for less time. Students, who spend more time in the practice room, may simply have more information to report about their strategies. By contrast, professional musicians may spend more time in activities such as rehearsals, performances or teaching (Vellacott and Ballantyne, 2022). Thus, self-regulation eventually serves as a determining factor for individuals to be able to sustain their artistic activities. Studies that measure SRL and time management in advanced musicians portray improvements in time management and enhanced practice efficiency as the main outcomes (Kim, 2010; Clark and Williamon, 2011; Miksza, 2015; Pike, 2017; López-Íñiguez and McPherson, 2020).

The Expertise variable categorized participants into Students, Pre-Professionals, and Professionals. ANOVA analysis identifies significant differences in the Personal Resources dimension, with Professionals obtaining higher scores than Pre-professionals. On the other hand, in the External Resources dimension, Students scored higher than Professionals.

The same differences emerged when musicians were asked to report the number of years since their first public performance. Musicians reporting more years since their first public performance scored higher in the Personal Resources dimension and lower in the External Resources dimension. Similarly, in terms of age, older musicians scored higher in the Personal Resources dimension and lower in the External Resources dimension. Bonneville-Roussy and Bouffard (2015) describe how older participants deploy deliberate practice strategies more frequently than younger participants. These findings suggest that, as musicians gain in experience, their metacognitive processes become more relevant than the social factors of their performance.

The items included in the External Resources dimension encompass processes such as seeking help from others (teachers, peers, composers, musicologists, and specialists) as well as actively searching for other sources of information able to support daily practice, such as books, recordings, videos, the Internet, and social media. In the 2002 article by McPherson and Zimmerman, the first to adapt Zimmerman’s cyclical model to music learning, the authors state they did not find any mention of seeking external resources in the literature on musical practice (McPherson and Zimmerman, 2002). Two decades later, music practice research has advanced (How et al., 2022) and therefore enables discussion of these results. Over the years, other studies have reported that professional musicians rely less on external assistance when preparing for performances, in comparison with students, even among advanced musicians from diverse cultural backgrounds who not only received different musical education but also face different job markets (Nielsen, 2004; Dos Santos and Gerling, 2011; Araújo, 2016; Volioti and Williamon, 2017).

On the other hand, when approaching undergraduate students, studies examining the practices of pre-service music teachers obtain results that portray how more advanced students on this study program employ more help-seeking processes than their peers during the early years of the program (Boon, 2020; Kaleli, 2021). Similarly, microanalysis studies register the greater use of external resources among undergraduate participants with higher music performance scores (Miksza et al., 2018; McPherson et al., 2019; Osborne et al., 2021). This suggests that, even though professionals report minimal usage of strategies related to social factors, this behavior is adopted by students with higher levels of performance evaluation in keeping with how aspiring musicians can benefit from seeking external resources during their practice sessions. This proactive behavior enables them to engage in “modeling, listening, and critical appraisal” (Ritchie and Williamon, 2013) and engage in positive reinforcement through exchanging knowledge with peers (Dos Santos Silva et al., 2023; Liu, 2023b), which are crucial for their growth and attainment of performance excellence, particularly during the learning phase.

The results of this study should be considered in light of its limitations. The sample consisted of volunteers, which may affect the generalizability of the findings and may not fully encompass the variety existing in the population studied. Nevertheless, data anonymity may have mitigated potential sample bias.

Furthermore, SRL processes in descriptive research may reach a broader population but may not accurately reflect actual practice behaviors. Recent studies employing the same questionnaire in structured interviews have indicated that conceptions of SRL processes, such as goal-setting and environment structuring, diverge considerably among advanced musicians (Silva and Fiorini, 2021). Other studies have combined quantitative scales with the observation of SRL processes as they occur, for example in microanalysis studies (Miksza et al., 2018; McPherson et al., 2019; Osborne et al., 2021). Future research might combine large sample surveys with observation applied to a sample subset. Moreover, follow-up studies using quantitative scales could efficiently measure the maintenance of SRL behaviors learned through intervention over time.

## 5 Conclusion

The purpose of our study was to evaluate the reliability of a self-regulation measurement scale in Portuguese. Additionally, we sought to gather current information about the practice habits of advanced musicians who study and work in two Portuguese-speaking countries. Our two first hypotheses were confirmed: the EFA results organized the same items into the three dimensions as Araújo (2016) first study, suggesting that the questionnaire is robust for assessing SRL processes. We may also report that there were no significant differences in SRL scores based on gender, nationality or musical instrument. Participants who declared practicing for more time scored higher in the Practice Organization dimension across both variables (hours per day and days per week). In the External Resources dimension, musicians who declared practicing every day of the week scored higher (than all the other categories). However, participants who reported practicing more hours per day then scored lower in this dimension, and that partially confirms hypothesis 3. Lastly, the fourth hypothesis was also partially confirmed. More experienced musicians scored higher in the Personal Resources dimension but lower in the External Resources dimension, based on expertise, age, and years since their first public performance.

The results of this research suggest that SRL constitutes a set of processes that musicians acquire throughout their learning journey and that these interlink with a significant amount of practice time. As these processes become internalized, practicing becomes more efficient and the time required to achieve performance goals decreases. Similarly, the search for assistance and external resources is an expected behavior in the professional development of musicians. As they attain higher levels of professional performance, personal resources surpass their recourse to external factors.

Developing and validating questionnaires tailored to the specificities of music practice should be encouraged as this may improve music teacher diagnosis of just which SRL dimensions their students need to consider most as well as keeping track of positive changes in SRL behaviors. In the present study, we provided psychometric evidences to this instrument; therefore, new studies should be conducted to establish norms that will be used to contextualize individual scores on this test. Future studies may use the questionnaire to collect empirical results and compare scores with new samples in the context of Brazil and Portugal, concerning the total table.

In small samples, from which data may not be generalized, the questionnaire is especially helpful as it may encourage advanced students to stop and reflect on their practice habits (Silva and Fiorini, 2021). Furthermore, future research might further assess the consistency of this SRL measuring scale for the learning processes undertaken by beginner and intermediate musicians.

## Data availability statement

The datasets presented in this article are not readily available because the raw data supporting the conclusions of this article will be available after the full research is completed. Requests to access the datasets should be directed to camillasilvamusica@gmail.com.

## Ethics statement

The studies involving humans were approved by UNICAMP–PRÓ-REITORIA DE PESQUISA DA UNIVERSIDADE ESTADUAL DE CAMPINAS–Comitê de Ética em Pesquisa em Ciências Humanas–CHS/UNICAMP. CAAE: 09319219.8.0000.8142. The studies were conducted in accordance with the local legislation and institutional requirements. The participants provided their on-line informed consent to participate in this study.

## Author contributions

CS: Data curation, Formal analysis, Funding acquisition, Investigation, Methodology, Software, Validation, Writing – original draft, Writing – review & editing, Conceptualization. MA: Conceptualization, Data curation, Investigation, Methodology, Software, Writing – review & editing, Writing – original draft. HM: Funding acquisition, Supervision, Writing – review & editing, Writing – original draft.
