# Supplementary material for: Attitudes in music practice: a survey exploring the self-regulated learning processes of advanced Brazilian and Portuguese musicians
Source: Front Psychol. 2024 Jan 31;15:1324100. doi: 10.3389/fpsyg.2024.1324100 (PMC10864437; doi:10.3389/fpsyg.2024.1324100)
Supplement: Supplementary file 1 [file Data_Sheet_1.docx]

Supplementary Material

# Supplementary Data: Survey: English Version

Attitudes in Music Practice

**Demographical information**

1. Age: ____

2. Gender:

| Male |  |
| --- | --- |
| Female |  |
| Non-binary |  |
|  |  |

3. Nationality: ____________________________

4. Academic qualifications (Please indicate your most advanced academic qualification)

| Academic qualification | Start year | Conclusion year |
| --- | --- | --- |
| Primary level |  |  |
| Secondary level |  |  |
| Bachelor degree |  |  |
| Post-graduate certificate or diploma |  |  |
| Masters degree |  |  |
| Doctoral qualification |  |  |
| Other: (please specify)_______ |  |  |

**Musical instrument**

6. What is your main musical instrument (singers please answer ‘voice’): ______________________________

7. Please indicate how old you were when you started taking instrumental or vocal lessons: _____ years

8. Performance experience
How many years have you been performing in public (from your first public performance)? ____ years

**Practice frequency and performances**

9. Concerts per year:
Please indicate the average number of concerts per year in which you have an important role (consider school auditions, public performances, chamber music performances, solos with an orchestra, among others):

| Less than 10 |  | Between 10 - 20 |  | Between 21 - 30 |  | Between 31 - 40 |  | More than 40 |  |
| --- | --- | --- | --- | --- | --- | --- | --- | --- | --- |

10. Practice: hours
How many hours do you currently practice per day (average)?

| No more than 1h |  | Between 1h - 2h |  | Between 2h - 3h |  | Between 3h - 4h |  | More than 4h |  |
| --- | --- | --- | --- | --- | --- | --- | --- | --- | --- |

11. Practice: days
How many days do you currently practice per week (average)?

| 1 - 2 days |  | 3 - 4 days |  | 5 - 6 days |  | Every day |  |
| --- | --- | --- | --- | --- | --- | --- | --- |

12. Have you ever been awarded a prize in a musical performance competition?

| Yes |  |
| --- | --- |
| No |  |

12.1 If you answered ‘yes’, please indicate how many prizes you received: _____
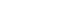


**ATTITUDES TOWARDS THE PRACTICE PROCESS**

**4=Agree**

Please indicate your response to the following statements:

In my practice:

**1=Never 2=Rarely 3=Sometimes 4=Often 5=Always**

| I set goals for my practice sessions | 1 | 2 | 3 | 4 | 5 |
| --- | --- | --- | --- | --- | --- |
| I set short-term goals (minutes, hours, days) | 1 | 2 | 3 | 4 | 5 |
| I set long-term goals (weeks, months, years) | 1 | 2 | 3 | 4 | 5 |
| I set specific goals for my practice sessions | 1 | 2 | 3 | 4 | 5 |
| I understand that my goals are challenging | 1 | 2 | 3 | 4 | 5 |

In my practice:

**1=Completely disagree 2=Disagree 3=Neither agree nor disagree 4=Agree 5=Completely agree**

| I use specific strategies related to my practice goals | 1 | 2 | 3 | 4 | 5 |
| --- | --- | --- | --- | --- | --- |
| I am aware of the strategies that I use during practice. | 1 | 2 | 3 | 4 | 5 |
| I use strategies that have been effective in the past. | 1 | 2 | 3 | 4 | 5 |
| I know when and in which contexts my strategies will be most effective | 1 | 2 | 3 | 4 | 5 |
| I understand the nature and demands of my musical activities that my goals are challenging | 1 | 2 | 3 | 4 | 5 |
| I know what I must do in order to complete my musical activities satisfactorily | 1 | 2 | 3 | 4 | 5 |

In my practice:

**1=Never 2=Rarely 3=Sometimes 4=Often 5=Always**

| I plan the order of the activities of my practice sessions | 1 | 2 | 3 | 4 | 5 |
| --- | --- | --- | --- | --- | --- |
| I plan the time of my practice sessions | 1 | 2 | 3 | 4 | 5 |
| I organize the physical environment of my practice sessions | 1 | 2 | 3 | 4 | 5 |
| I evaluate the progress made towards my goals | 1 | 2 | 3 | 4 | 5 |
| I seek information from several sources (books, CDs, videos, internet, biographies, arts, etc.) to support my study | 1 | 2 | 3 | 4 | 5 |
| I request help from others (teachers, peers, composers, musicologists and specialists) | 1 | 2 | 3 | 4 | 5 |

Reflecting about my own characteristics as a performer, I believe that:

**1=Completely disagree 2=Disagree 3=Neither agree nor disagree 5=Completely agree**

| I am able to achieve my practice goals satisfactorily | 1 | 2 | 3 | 4 | 5 |
| --- | --- | --- | --- | --- | --- |
| I cannot reach my practice goals without the support of some external factors (peers, teachers, materials, environment) | 1 | 2 | 3 | 4 | 5 |
| I understand my strengths and weaknesses | 1 | 2 | 3 | 4 | 5 |
| I practice in order to improve my musical skills | 1 | 2 | 3 | 4 | 5 |
| I practice in order to achieve high ratings (e.g. grades) and positive feedback | 1 | 2 | 3 | 4 | 5 |

# Supplementary Tables

|  |  | F1 | F2 |
| --- | --- | --- | --- |
| 1 | I set goals for my practice sessions | 0.784 |  |
| 2 | I set short term goals (minutes, hours, days) | 0.600 |  |
| 3 | I set long-term goals (weeks, months, years) | 0.475 |  |
| 4 | I set specific goals for my practice sessions | 0.792 |  |
| 5 | I understand my goals are challenging | 0.470 |  |
| 6 | I use specific strategies related to my practice goals | **0.321** | **0.527** |
| 12 | I plan the order of the activities of my practice sessions | 0.745 |  |
| 13 | I plan the time of my practice sessions | 0.715 |  |
| 14 | I organize the physical environment of my practice sessions | 0.527 |  |
| 15 | I evaluate the progress made towards my goals | 0.628 |  |
| 16 | I seek information from several sources (books, CDs, videos, internet, biographies, arts, etc.) to support my study | 0.309 |  |
| 17 | I request help from others (teachers, peers, composers, musicologists and specialists) | **0.507** | **-0.305** |
| 19 | I cannot reach my practice goals without the support of some external factors (peers, teachers, materials, environment) | 0.412 |  |
| 21 | I practice in order to improve my musical skills | 0.472 |  |
| 22 | I practice in order to achieve high ratings (e.g. grades) and positive feedback | 0.365 |  |
| 7 | I am aware of the strategies that I use during practice |  | 0.806 |
| 8 | I use strategies that have been effective in the past |  | 0.487 |
| 9 | I know when and in which contexts my strategies will be most effective |  | 0.866 |
| 10 | I understand the nature and demands of my musical activities |  | 0.729 |
| 11 | I know what I must do in order to complete my musical activities satisfactorily |  | 0.855 |
| 18 | I am able to achieve my practice goals satisfactorily |  | 0.480 |
| 20 | I understand my strengths and weaknesses |  | 0.627 |

## Supplementary Table 1. EFA 2-factor model

## Supplementary Table 2. EFA 3-factor model (item 6 - crossloading)

|  |  | F1 | F2 | F3 |
| --- | --- | --- | --- | --- |
| 1 | I set goals for my practice sessions | 0.963 |  |  |
| 2 | I set short term goals (minutes, hours, days) | 0.726 |  |  |
| 3 | I set long-term goals (weeks, months, years) | 0.380 |  |  |
| 4 | I set specific goals for my practice sessions | 0.953 |  |  |
| 12 | I plan the order of the activities of my practice sessions | 0.709 |  |  |
| 13 | I plan the time of my practice sessions | 0.703 |  |  |
| 14 | I organize the physical environment of my practice sessions | 0.451 |  |  |
| 15 | I evaluate the progress made towards my goals | 0.636 |  |  |
| 6 | I use specific strategies related to my practice goals | 0.364 | **0.498** |  |
| 7 | I am aware of the strategies that I use during practice |  | 0.802 |  |
| 8 | I use strategies that have been effective in the past |  | 0.496 |  |
| 9 | I know when and in which contexts my strategies will be most effective |  | 0.841 |  |
| 10 | I understand the nature and demands of my musical activities |  | 0.786 |  |
| 11 | I know what I must do in order to complete my musical activities satisfactorily |  | 0.846 |  |
| 18 | I am able to achieve my practice goals satisfactorily |  | 0.465 |  |
| 20 | I understand my strengths and weaknesses |  | 0.666 |  |
| 05 | I understand my goals are challenging |  |  | 0.301 |
| 16 | I seek information from several sources (books, CDs, videos, internet, biographies, arts, etc.) to support my study |  |  | 0.417 |
| 17 | I request help from others (teachers, peers, composers, musicologists and specialists) |  |  | 0.707 |
| 19 | I cannot reach my practice goals without the support of some external factors (peers, teachers, materials, environment) |  |  | 0.634 |
| 21 | I practice in order to improve my musical skills |  |  | 0.451 |
| 22 | I practice in order to achieve high ratings (e.g. grades) and positive feedback |  |  | 0.321 |

## Supplementary Table 3. Gender: T-test - descriptive statistics

| Dimension | | | Statistics |  | Bootstrap^a^ | |
| --- | --- | --- | --- | --- | --- | --- |
|  |  |  |  | Standard Error | BCa 95% Confidence Interval | |
|  |  |  |  |  | Lower | Upper |
| Practice Organization | Masc | *N* | 162 |  |  |  |
|  |  | Mean | 29.64 | .51 | 28.61 | 30.68 |
|  |  | SD | 6.38 | .33 | 5.76 | 6.91 |
|  | Fem | *N* | 132 |  |  |  |
|  |  | Mean | 30.97 | .46 | 29.74 | 31.58 |
|  |  | SD | 5.33 | .36 | 4.66 | 5.99 |
| Personal Resources | Masc | *N* | 162 |  |  |  |
|  |  | Mean | 28.92 | .31 | 28.28 | 29.56 |
|  |  | SD | 3.98 | .25 | 3.53 | 4.39 |
|  | Fem | *N* | 132 |  |  |  |
|  |  | Mean | 28.42 | .33 | 27.80 | 29.06 |
|  |  | SD | 3.91 | .24 | 3.45 | 4.36 |
| External Resources | Masc | *N* | 162 |  |  |  |
|  |  | Mean | 18.80 | .27 | 18.23 | 19.34 |
|  |  | SD | 3.43 | .16 | 3.11 | 3.68 |
|  | Fem | *N* | 132 |  |  |  |
|  |  | Mean | 19.14 | .25 | 18.63 | 19.63 |
|  |  | SD | 2.93 | .19 | 2.57 | 3.25 |
| Total | Masc | *N* | 162 |  |  |  |
|  |  | Mean | 77.35 | 0.89 | 75.48 | 79.21 |
|  |  | SD | 11.19 | 0.63 | 10.02 | 12.15 |
|  | Fem | *N* | 132 |  |  |  |
|  |  | Mean | 78.23 | 0.71 | 76.79 | 79.67 |
|  |  | SD | 8.40 | 0.48 | 7.58 | 9.17 |

Unless otherwise noted, bootstrap results are based on 1000 bootstrap samples

## Supplementary Table 4. Nationality: T-test - descriptive statistics

| Dimension | | | Statistics |  | Bootstrap^a^ | |
| --- | --- | --- | --- | --- | --- | --- |
|  |  |  |  | Standard Error | BCa 95% Confidence Interval | |
|  |  |  |  |  | Lower | Upper |
| Practice Organization | Brazilian | *N* | 202 |  |  |  |
|  |  | Mean | 30.06 | .43 | 29.18 | 30.95 |
|  |  | SD | 6.19 | .32 | 5.61 | 6.70 |
|  | Portuguese | *N* | 95 |  |  |  |
|  |  | Mean | 30.18 | .52 | 29.19 | 31.23 |
|  |  | SD | 5.33 | .34 | 4.67 | 5.90 |
| Personal Resources | Brazilian | *N* | 202 |  |  |  |
|  |  | Mean | 28.49 | .28 | 27.93 | 29.03 |
|  |  | SD | 4.08 | .22 | 3.66 | 4.46 |
|  | Portuguese | *N* | 95 |  |  |  |
|  |  | Mean | 29.11 | .37 | 28.36 | 29.82 |
|  |  | SD | 3.58 | .22 | 3.20 | 3.90 |
| External Resources | Brazilian | *N* | 202 |  |  |  |
|  |  | Mean | 19.04 | .24 | 18.54 | 19.48 |
|  |  | SD | 3.40 | .15 | 3.10 | 3.66 |
|  | Portuguese | *N* | 95 |  |  |  |
|  |  | Mean | 18.74 | .28 | 18.15 | 19.26 |
|  |  | SD | 2.75 | .20 | 2.40 | 3.08 |
| Total | Brazilian | *N* | 202 |  |  |  |
|  |  | Mean | 77.59 | 0.72 | 76.04 | 78.98 |
|  |  | SD | 10.46 | 0.57 | 9.48 | 11.41 |
|  | Portuguese | *N* | 95 |  |  |  |
|  |  | Mean | 78.02 | 0.88 | 76.46 | 79.72 |
|  |  | SD | 8.92 | 0.54 | 7.96 | 9.74 |

## Supplementary Table 5. Musical Instrument: descriptive statistics

|  | | Practice Organization | | | Personal Resources | | | External Resources | | | Total | | |
| --- | --- | --- | --- | --- | --- | --- | --- | --- | --- | --- | --- | --- | --- |
|  | | | BCa 95% Confidence Interval | |  | BCa 95% Confidence Interval | |  | BCa 95% Confidence Interval | |  | BCa 95% Confidence Interval | |
| Instrument | | Statistic | Lower | Upper | Statistic | Lower | Upper | Statistic | Lower | Upper | Statistic | Lower | Upper |
| Plucked Strings | *N* | 149 |  |  | 148 |  |  | 149 |  |  | 148 |  |  |
|  | Mean | 30.19 | 29.17 | 31.14 | 28.67 | 28.07 | 29.27 | 19.03 | 18.47 | 19.57 | 77.85 | 76.25 | 79.39 |
|  | SD | 5.91 | 5.29 | 6.43 | 3.94 | 3.45 | 4.38 | 3.45 | 3.11 | 3.74 | 10.09 | 8.93 | 11.24 |
| Keyboards | *N* | 32 |  |  | 32 |  |  | 32 |  |  | 32 |  |  |
|  | Mean | 30.00 | 27.69 | 32.26 | 28.66 | 27.23 | 30.00 | 17.97 | 17.22 | 18.73 | 76.63 | 73.07 | 80.18 |
|  | SD | 6.37 | 4.88 | 7.36 | 3.86 | 3.28 | 4.23 | 2.42 | 1.94 | 2.79 | 9.74 | 7.63 | 11.33 |
| Bow Strings | *N* | 38 |  |  | 38 |  |  | 38 |  |  | 38 |  |  |
|  | Mean | 29.45 | 27.69 | 32.26 | 27.63 | 26.32 | 28.78 | 19.47 | 18.63 | 20.42 | 76.55 | 73.83 | 79.38 |
|  | SD | 6.37 | 4.88 | 7.36 | 3.67 | 2.67 | 4.45 | 2.78 | 2.37 | 3.10 | 9.17 | 7.46 | 10.60 |
| Voice | *N* | 24 |  |  | 24 |  |  | 24 |  |  | 24 |  |  |
|  | Mean | 30.17 | 27.62 | 32.60 | 28.58 | 26.80 | 30.29 | 18.83 | 17.44 | 20.10 | 77.58 | 72.88 | 81.82 |
|  | SD | 6.08 | 3.86 | 7.52 | 4.48 | 3.41 | 5.26 | 3.27 | 2.57 | 3.77 | 11.05 | 7.27 | 13.86 |
| Wind | *N* | 46 |  |  | 46 |  |  | 46 |  |  | 46 |  |  |
|  | Mean | 31.17 | 29.59 | 32.86 | 29.57 | 28.33 | 30.81 | 19.26 | 18.27 | 20.30 | 80.00 | 77.13 | 82.73 |
|  | SD | 5.73 | 4.94 | 6.39 | 3.97 | 3.19 | 4.63 | 3.34 | 2.82 | 3.78 | 10.21 | 8.82 | 11.39 |
| Total | *N* | 289 |  |  | 288 |  |  | 289 |  |  | 288 |  |  |
|  | Mean | 30.23 | 29.52 | 30.98 | 28.67 | 28.23 | 29.12 | 18.99 | 18.63 | 19.37 | 77.86 | 76.70 | 79.06 |
|  | SD | 5.91 | 5.46 | 6.32 | 3.95 | 3.62 | 4.27 | 3.24 | 3.02 | 3.45 | 10.03 | 9.19 | 10.83 |

## Supplementary Table 6. Practice hours per day: descriptive statistics

|  |  |  | | Practice Organization | | | Personal Resources | | | External Resources | | | Total | | |
| --- | --- | --- | --- | --- | --- | --- | --- | --- | --- | --- | --- | --- | --- | --- | --- |
|  |  |  | |  | BCa 95% Confidence Interval | |  | BCa 95% Confidence Interval | |  | BCa 95% Confidence Interval | |  | BCa 95% Confidence Interval | |
| Practice hours per day | | | Statistic | | Lower | Upper | Statistic | Lower | Upper | Statistic | Lower | Upper | Statistic | Lower | Upper |
| Less than 1h | *N* | | 47 | |  |  | 47 | 36 |  | 47 |  |  | 47 |  |  |
|  | Mean | | 28.55 | | 26.80 | 30.25 | 27.49 | 26.11 | 28.71 | 17.09 | 16.08 | 18.18 | 73.13 | 69.62 | 76.53 |
|  | SD | | 6.05 | | 4.92 | 7.01 | 4.70 | 3.787 | 5.51 | 3.49 | 2.95 | 3.95 | 11.82 | 9.73 | 13.41 |
| 1h - 2h | *N* | | 83 | |  |  | 83 |  |  | 83 |  |  | 83 |  |  |
|  | Mean | | 29.17 | | 27.88 | 30.41 | 28.48 | 27.67 | 29.24 | 18.55 | 17.95 | 19.16 | 76.20 | 73.82 | 78.47 |
|  | SD | | 6.26 | | 5.32 | 7.10 | 3.77 | 3.17 | 4.29 | 3.03 | 2.67 | 3.33 | 9.90 | 8.41 | 11.06 |
| 2h - 3h | *N* | | 80 | |  |  | 80 |  |  | 80 |  |  | 80 |  |  |
|  | Mean | | 30.05 | | 28.51 | 31.35 | 29.00 | 28.17 | 29.73 | 19.44 | 18.73 | 20.23 | 78.49 | 76.45 | 80.40 |
|  | SD | | 5.75 | | 5.00 | 6.48 | 3.91 | 3.25 | 4.54 | 3.34 | 2.87 | 3.75 | 9.14 | 8.07 | 10.17 |
| 3h - 4h | *N* | | 59 | |  |  | 57 |  |  | 59 |  |  | 57 |  |  |
|  | Mean | | 31.53 | | 30.02 | 32.95 | 29.21 | 28.27 | 30.18 | 20.14 | 19.45 | 20.77 | 80.61 | 78.53 | 82.73 |
|  | SD | | 5.08 | | 4.20 | 5.84 | 3.58 | 3.02 | 4.11 | 2.70 | 2.32 | 3.00 | 8.59 | 7.27 | 9.72 |
| More than 4h | *N* | | 30 | |  |  | 30 |  |  | 30 |  |  | 30 |  |  |
|  | Mean | | 32.77 | | 30.74 | 34.58 | 29.30 | 28.00 | 30.48 | 19.60 | 18.64 | 20.63 | 81.67 | 78.63 | 84.76 |
|  | SD | | 5.69 | | 4.26 | 6.82 | 3.54 | 2.94 | 4.00 | 2.52 | 1.98 | 2.94 | 8.56 | 7.11 | 9.64 |
| Total | *N* | | 299 | |  |  | 297 |  |  | 299 |  |  | 297 |  |  |
|  | Mean | | 30.13 | | 29.49 | 30.81 | 28.69 | 28.29 | 29.05 | 18.98 | 18.61 | 19.31 | 77.73 | 76.52 | 78.96 |
|  | SD | | 5.93 | | 5.46 | 6.41 | 3.93 | 3.57 | 4.32 | 3.22 | 2.99 | 3.42 | 9.98 | 9.21 | 10.70 |

## Supplementary Table 7. Practice hours per day: ANOVA Post-hoc tests with Bonferroni correction and CI through Bootstrapping

|  |  | Practice Organization | | | | External Resources | | | | | Total | | | | |
| --- | --- | --- | --- | --- | --- | --- | --- | --- | --- | --- | --- | --- | --- | --- | --- |
|  |  |  | | BCa 95% Confidence Interval | |  | | | BCa 95% Confidence Interval | |  | | | BCa 95% Confidence Interval | |
| (I) Practice hours per day | (J) Practice hours per day | Mean dif.  (I-J) | *p* | Lower | Upper | Mean dif.  (I-J) | | p | Lower | Upper | Mean dif.  (I-J) | | p | Lower | Upper |
| Less than 1h | 1h - 2h | -.615 | 1.000 | -3.031 | 1.779 | -1.469 | | .097 | -2.608 | -.255 | -3.077 | | .826 | -8.07 | 1.92 |
|  | 2h - 3h | -1.497 | 1.000 | -3.479 | .428 | -2.352* | | <.001 | -3.579 | -1.064 | -5.360* | | .028 | -10.39 | -.33 |
|  | 3h - 4h | -2.972 | .095 | -5.296 | -.915 | -3.050* | | <.001 | -4.240 | -1.715 | -7.486* | | .001 | -12.88 | -2.09 |
|  | More than 4h | -4.213* | .021 | -6.774 | -1.425 | -2.515* | | .006 | -3.883 | -1.141 | -8.539* | | .002 | -14.94 | -2.14 |
| 1h - 2h | Less than 1h | .615 | 1.000 | -1.657 | 2.841 | 1.469 | | .097 | .251 | 2.714 | 3.077 | | .826 | -1.92 | 8.07 |
|  | 2h - 3h | -.881 | 1.000 | -2.716 | .811 | -.883 | | .692 | -1.83 9 | -.004 | -2.283 | | 1.000 | -6.57 | 2.01 |
|  | 3h - 4h | -2.357 | .181 | -4.129 | -.677 | -1.581* | | .029 | -2.478 | -.653 | -4.409 | | .085 | -9.12 | .30 |
|  | More than 4h | -3.598* | .040 | -6.042 | -1.015 | -1.046 | | 1.000 | -2.135 | .051 | -5.462 | | .085 | -11.29 | .37 |
| 2h - 3h | Less than 1h | 1.497 | 1.000 | -.950 | 3.977 | 2.352* | | <.001 | 1.079 | 3.571 | 5.360* | | .028 | .33 | 10.39 |
|  | 1h - 2h | .881 | 1.000 | -1.021 | 2.942 | .883 | | .692 | -.147 | 1.885 | 2.283 | | 1.000 | -2.01 | 6.57 |
|  | 3h - 4h | -1.475 | 1.000 | -3.400 | .454 | -.698 | | 1.000 | -1.740 | .396 | -2.127 | | 1.000 | -6-87 | 2.62 |
|  | More than 4h | -2.717 | .301 | -5.333 | -.074 | -.163 | | 1.000 | -1.384 | 1.044 | -3.179 | | 1.000 | -9.04 | 2.68 |
| 3h - 4h | Less than 1h | 2.972 | .095 | .872 | 5.377 | 3.050* | | <.001 | 1.715 | 4.241 | 7.486* | | .001 | 2.09 | 12.88 |
|  | 1h - 2h | 2.357 | .181 | .193 | 4.580 | 1.581* | | .029 | .642 | 2.510 | 4.409 | | .085 | -.30 | 9.12 |
|  | 2h - 3h | 1.475 | 1.000 | -.465 | 3.417 | .698 | | 1.000 | -.246 | 1.623 | 2.127 | | 1.000 | -2.62 | 6.87 |
|  | More than 4h | -1.241 | 1.000 | -3.854 | 1.413 | .536 | 1.000 | | -.571 | 1.609 | -1.053 | 1.000 | | -7.23 | 5.12 |
| More than 4h | Less than 1h | 4.213* | -021 | 1.538 | 6.709 | 2.515* | .006 | | 1.122 | 3.901 | 8.539* | .002 | | 2.14 | 14.94 |
|  | 1h - 2h | 3.598* | .040 | 1.024 | 6.036 | 1.046 | 1.000 | | -.124 | 2.238 | 5.462 | .085 | | -.37 | 11.29 |
|  | 2h - 3h | 2.717 | .301 | .438 | 4.895 | .163 | 1.000 | | -.986 | 1.320 | 3.179 | 1.000 | | -2.68 | 9.04 |
|  | 3h - 4h | 1.241 | 1.000 | -1.100 | 3.456 | -.536 | 1.000 | | -1.882 | .828 | 1.053 | 1.000 | | -5.12 | 7.23 |

## Supplementary Table 8. Practice days per week: descriptive statistics

|  |  |  | | Practice Organization | | | Personal Resources | | | External Resources | | | Total | | |
| --- | --- | --- | --- | --- | --- | --- | --- | --- | --- | --- | --- | --- | --- | --- | --- |
|  |  |  | |  | BCa 95% Confidence Interval | |  | BCa 95% Confidence Interval | |  | BCa 95% Confidence Interval | |  | BCa 95% Confidence Interval | |
| Practice days per week | | | Statistic | | Lower | Upper | Statistic | Lower | Upper | Statistic | Lower | Upper | Statistic | Lower | Upper |
| 1 - 2 days | *N* | | 40 | |  |  | 40 |  |  | 40 |  |  | 40 |  |  |
|  | Mean | | 26.88 | | 24.95 | 28.84 | 27.60 | 26.09 | 29.02 | 16.95 | 15.98 | 17.84 | 71.43 | 67.83 | 75.00 |
|  | SD | | 6.51 | | 5.23 | 7.58 | 4.88 | 3.88 | 5.73 | 3.08 | 2.54 | 3.48 | 11.31 | 8.91 | 13.32 |
| 3 - 4 days | *N* | | 70 | |  |  | 70 |  |  | 70 |  |  | 70 | 70 | 97 |
|  | Mean | | 29.54 | | 28.07 | 30.97 | 28.91 | 27.89 | 29.91 | 18.47 | 17.79 | 19.10 | 76.93 | 74.45 | 79.49 |
|  | SD | | 5.81 | | 4.92 | 6.57 | 4.24 | 3.66 | 4.73 | 2.82 | 2.44 | 3.17 | 10.26 | 8.88 | 11.44 |
| 5 - 6 days | *N* | | 112 | |  |  | 112 |  |  | 112 |  |  | 112 |  |  |
|  | Mean | | 30.83 | | 29.65 | 31.95 | 28.60 | 27.91 | 29.22 | 19.28 | 18.69 | 19.87 | 78.71 | 77.07 | 80.31 |
|  | SD | | 5.74 | | 5.04 | 6.45 | 3.52 | 3.09 | 3.89 | 3.04 | 2.70 | 3.34 | 9.14 | 8.07 | 10.17 |
| Every day | *N* | | 77 | |  |  | 77 |  |  | 77 |  |  | 77 |  |  |
|  | Mean | | 31.35 | | 30.14 | 32.56 | 29.19 | 28.36 | 29.97 | 20.05 | 19.22 | 20.88 | 80.39 | 78.30 | 82.35 |
|  | SD | | 5.39 | | 4.61 | 6.16 | 3.61 | 3.01 | 4.16 | 3.37 | 2.77 | 3.85 | 9.12 | 7.86 | 10.33 |
| Total | *N* | | 299 | | . | . | 297 | . | . | 299 | . | . | 297 | . | . |
|  | Mean | | 30.13 | | 29.42 | 30.86 | 28.69 | 28.26 | 29.10 | 18.98 | 18.59 | 19.37 | 77.73 | 76.54 | 78.93 |
|  | SD | | 5.93 | | 5.46 | 6.46 | 3.93 | 3.60 | 4.24 | 3.22 | 2.99 | 3.41 | 9.98 | 9.18 | 10.76 |

## Supplementary Table 9. Practice days per week: ANOVA Post-hoc tests with Bonferroni correction and CI through Bootstrapping

|  |  | Practice Organization | | | | External Resources | | | | Total | | | |
| --- | --- | --- | --- | --- | --- | --- | --- | --- | --- | --- | --- | --- | --- |
|  |  |  | | BCa 95% Confidence Interval | |  | | BCa 95% Confidence Interval | |  | | BCa 95% Confidence Interval | |
| (I) Days of practice per week | (J) Days of practice per week | Mean Dif.  (I-J) | *p* | Lower | Upper | Mean Dif.  (I-J) | p | Lower | Upper | Mean Dif.  (I-J) | p | Lower | Upper |
| 1 - 2 days | 3 - 4 days | -2-668 | .124 | -5.121 | -.361 | -1.521 | .082 | -2.678 | -.474 | -5.504* | .026 | -9.749 | -1.537 |
|  | 5 - 6 days | -3.955* | .001 | -6.134 | -1.859 | -2.327* | <.001 | -3.423 | -1.246 | -7.280* | <.001 | -11.073 | -3.301 |
|  | Every day | -4.476* | <.001 | -6.613 | -2.247 | -3.102* | <.001 | -4.366 | -2.061 | -8.962* | <.001 | -13.196 | -4.758 |
| 3 - 4 days | 1 - 2 days | 2.668 | .124 | .390 | 5.114 | 1.521 | .082 | .444 | 2.719 | 5.504* | .026 | -1.249 | 10-315 |
|  | 5 - 6 days | -1.287 | .870 | -2.922 | .390 | -.805 | .530 | -1.649 | .063 | -1.777 | 1.000 | -4.819 | 1.314 |
|  | Every day | -1.808 | .356 | -3.753 | -.004 | -1.581* | .013 | -2.560 | -.651 | -3.458 | .191 | -6.863 | -.042 |
| 5 - 6 days | 1 - 2 days | 3.955* | .001 | 1.785 | 6.178 | 2.327* | <.001 | 1.188 | 3.503 | 7.280* | <.001 | 3.361 | 11.041 |
|  | 3 - 4 days | 1.287 | .870 | -.556 | 3.023 | .805 | .530 | -.102 | 1.661 | 1.777 | 1.000 | -1.005 | 4.552 |
|  | Every day | -.520 | 1.000 | -2.139 | 1.034 | -.775 | .548 | -1.687 | .123 | -1.681 | 1.000 | -4.500 | .925 |
| Every day | 1 - 2 days | 4.476* | <.001 | 1.996 | 6.753 | 3.102* | <.001 | 1.888 | 4.544 | 8.962* | <.001 | 4.902 | 13.016 |
|  | 3 - 4 days | 1.808 | .356 | .075 | 3.652 | 1.581* | .013 | .615 | 2.609 | 3.458 | .191 | .494 | 6.236 |
|  | 5 - 6 days | .520 | 1.000 | -.964 | 2.089 | .775 | .548 | -.232 | 1.798 | 1.681 | 1.000 | -.908 | 4.450 |

## Supplementary Table 10. Expertise: descriptive statistics

|  |  |  | | Practice Organization | | | Personal Resources | | | External Resources | | | Total | | |
| --- | --- | --- | --- | --- | --- | --- | --- | --- | --- | --- | --- | --- | --- | --- | --- |
|  |  |  | |  | BCa 95% Confidence Interval | |  | BCa 95% Confidence Interval | |  | BCa 95% Confidence Interval | |  | BCa 95% Confidence Interval | |
| Expertise | | | Statistic | | Lower | Upper | Statistic | Lower | Upper | Statistic | Lower | Upper | Statistic | Lower | Upper |
| Student | *N* | | 58 | |  |  | 57 |  |  | 58 |  |  | 57 |  |  |
|  | Mean | | 30.57 | | 29.13 | 31.83 | 28.25 | 27.15 | 29.29 | 20.21 | 19.38 | 21.05 | 78.95 | 76.32 | 81.48 |
|  | SD | | 5.25 | | 4.56 | 5.79 | 4.20 | 3.38 | 4.93 | 3.31 | 2.82 | 3.71 | 10.09 | 8.68 | 11.29 |
| Pre-professional | *N* | | 80 | |  |  | 80 |  |  | 80 |  |  | 80 |  |  |
|  | Mean | | 28.84 | | 27.38 | 30.17 | 27.65 | 26.91 | 28.36 | 19.34 | 18.57 | 20.02 | 75.83 | 73.35 | 78.15 |
|  | SD | | 6.24 | | 5.24 | 7.06 | 3.62 | 3.14 | 4.07 | 3.34 | 2.91 | 3.73 | 10.49 | 8.89 | 11.83 |
| Professional | *N* | | 161 | |  |  | 160 |  |  | 161 |  |  | 160 |  |  |
|  | Mean | | 30.62 | | 29.71 | 31.44 | 29.36 | 28.74 | 29.97 | 18.35 | 17.93 | 18.84 | 78.25 | 76.82 | 79.74 |
|  | SD | | 5.93 | | 5.37 | 6.52 | 4.03 | 3.87 | 3.48 | 2.98 | 2.67 | 3.26 | 9.61 | 8.50 | 10.72 |
| Total | *N* | | 299 | |  |  | 297 |  |  | 299 |  |  | 297 |  |  |
|  | Mean | | 30.13 | | 29.47 | 30.81 | 28.69 | 28.25 | 29.10 | 18.98 | 18.56 | 19.33 | 77.73 | 76.62 | 78.86 |
|  | SD | | 5.93 | | 5.48 | 6.39 | 3.93 | 3.63 | 4.23 | 3.22 | 3.00 | 3.41 | 9.98 | 9.16 | 10.75 |

## Supplementary Table 11. Expertise: ANOVA Post-hoc tests with Bonferroni correction and CI through Bootstrapping

|  |  | Personal Resources | | | | External Resources | | | |  |
| --- | --- | --- | --- | --- | --- | --- | --- | --- | --- | --- |
|  |  |  | | BCa 95% Confidence Interval | |  | | BCa 95% Confidence Interval | |  |
| (I) Expertise | (J) Expertise | Mean Dif.  (I-J) | *p* | Lower | Upper | Mean Dif.  (I-J) | p | Lower | Upper | |
| Student | Pre-professional | .596 | 1.000 | -.838 | 1.992 | .869 | .332 | -.200 | 2.122 | |
|  | Professional | -1.117 | .188 | -2.382 | .090 | 1.853* | <.001 | .848 | 2.811 | |
| Pre-Professional | Student | -.596 | 1.000 | -1.886 | .737 | -.869 | .332 | -1.944 | .067 | |
|  | Professional | -1.713* | .004 | -2.611 | -.789 | .983 | .070 | -122 | 1.754 | |
| Professional | Student | 1.117 | .188 | -.002 | 2.352 | -1.853* | <.001 | -2.749 | -.955 | |
|  | Pre-professional | 1.713* | .004 | .728 | 2.649 | -.983 | .070 | -1.839 | -.054 | |

## Supplementary Table 12 Age: descriptive statistics

|  |  |  | | Practice Organization | | | Personal Resources | | | External Resources | | | Total | | |
| --- | --- | --- | --- | --- | --- | --- | --- | --- | --- | --- | --- | --- | --- | --- | --- |
|  |  |  | |  | BCa 95% Confidence Interval | |  | BCa 95% Confidence Interval | |  | BCa 95% Confidence Interval | |  | BCa 95% Confidence Interval | |
| Age range | | | Statistic | | Lower | Upper | Statistic | Lower | Upper | Statistic | Lower | Upper | Statistic | Lower | Upper |
| 18 - 25 | *N* | | 96 | |  |  | 95 |  |  | 96 |  |  | 95 |  |  |
|  | Mean | | 30.16 | | 29.15 | 31.12 | 27.88 | 27.12 | 28.64 | 20.28 | 19.62 | 20.86 | 78.27 | 76.12 | 80.35 |
|  | SD | | 5.17 | | 4.50 | 5.83 | 4.00 | 3.44 | 4.53 | 2.89 | 2.57 | 3.14 | 10.02 | 9-04 | 10-90 |
| 26 - 35 | *N* | | 104 | |  |  | 104 |  |  | 104 |  |  | 104 |  |  |
|  | Mean | | 29.03 | | 27.86 | 30.32 | 27.95 | 27.05 | 28.77 | 18.50 | 17.83 | 19.10 | 75.48 | 73.21 | 77.54 |
|  | SD | | 6.48 | | 5.72 | 7.18 | 4.09 | 3.54 | 4.58 | 3.27 | 2.88 | 3.61 | 11.11 | 9.49 | 12.52 |
| 36+ | *N* | | 99 | |  |  | 98 |  |  | 99 |  |  | 98 |  |  |
|  | Mean | | 31.27 | | 30.19 | 32.37 | 30.24 | 29.62 | 30.87 | 18.21 | 17.57 | 18.91 | 79.59 | 77.99 | 81.17 |
|  | SD | |  | |  |  | 3.20 | 2.86 | 3.48 | 3.12 | 2.73 | 3.46 | 8.17 | 7.17 | 9.00 |
| Total | *N* | | 299 | |  |  | 297 |  |  | 299 |  |  | 297 |  |  |
|  | Mean | | 30.13 | | 29.47 | 30.81 | 28.69 | 28.25 | 29.10 | 18.98 | 18.56 | 19.33 | 77.73 | 76.62 | 78.86 |
|  | SD | | 5.93 | | 5.48 | 6.39 | 3.93 | 3.64 | 4.23 | 3.22 | 3.00 | 3.41 | 9.98 | 9.16 | 10.75 |

## Supplementary Table 13. Age: ANOVA Post-hoc tests with Bonferroni correction and CI through Bootstrapping

|  |  | Practice Organization | | | | Personal Resources | | | | External Resources | | | | Total | | | |
| --- | --- | --- | --- | --- | --- | --- | --- | --- | --- | --- | --- | --- | --- | --- | --- | --- | --- |
|  |  |  | | BCa 95% Confidence Interval | |  | | BCa 95% Confidence Interval | |  | | BCa 95% Confidence Interval | |  | | BCa 95% Confidence Interval | |
| (I) Age range | (J) Age Range | Mean Dif.  (I-J) | *p* | Lower | Upper | Mean Dif.  (I-J) | *p* | Lower | Upper | Mean Dif.  (I-J) | *p* | Lower | Upper | Mean Dif. (I-J) | *p* | Lower | Upper |
| 18 - 25 | 26 -35 | 1.127 | .529 | -.361 | 2.653 | -.068 | 1.000 | -1.219 | 1.124 | 1.781* | <.001 | .916 | 2.747 | 2.793 | .141 | -.103 | 5.744 |
|  | 36+ | -1.116 | .557 | -2.745 | .561 | -2.361* | <.001 | -3.327 | -1.388 | 2.069* | <.001 | 1.225 | 2.853 | -1.318 | 1.000 | -4.051 | 1.319 |
| 26 - 35 | 18 - 25 | -1.127 | .529 | -2.777 | .502 | .068 | 1.000 | -1.063 | 1.185 | -1.781* | <.001 | -2.675 | -.922 | -2.793 | .141 | -5.772 | -.055 |
|  | 36+ | -2.244* | .021 | -3.939 | -.502 | -2.293* | <.001 | -3.318 | -1.342 | .288 | 1.000 | -.621 | 1.137 | -4.111* | .010 | -7.218 | -1.342 |
| 36+ | 18 - 25 | 1.116 | .557 | -.365 | 2.533 | 2.361* | <.001 | 1.348 | 3.386 | -2.069* | <.001 | -2.894 | -1.159 | 1.318 | 1.000 | -1.309 | 4.048 |
|  | 26 - 35 | 2.244* | .021 | -430 | 3.856 | 2.293* | <.001 | 1.335 | 3.328 | -.288 | 1.000 | -1.199 | .725 | 4.111* | .010 | 1.548 | 6.789 |

## Supplementary Table 14. Time since first public performance: descriptive statistics

|  |  |  | | Practice Organization | | | Personal Resources | | | External Resources | | | Total | | |
| --- | --- | --- | --- | --- | --- | --- | --- | --- | --- | --- | --- | --- | --- | --- | --- |
|  |  |  | |  | BCa 95% Confidence Interval | |  | BCa 95% Confidence Interval | |  | BCa 95% Confidence Interval | |  | BCa 95% Confidence Interval | |
| Years since first public concert | | | Statistic | | Lower | Upper | Statistic | Lower | Upper | Statistic | Lower | Upper | Statistic | Lower | Upper |
| 1 - 9 | *N* | | 48 | |  |  | 48 |  |  | 48 |  |  | 48 |  |  |
|  | Mean | | 30.06 | | 28.34 | 31.86 | 26.75 | 25.38 | 27.95 | 20.73 | 19.90 | 21.58 | 77.54 | 76.13 | 79.67 |
|  | SD | | 6.13 | | 5.13 | 6.95 | 4.47 | 3.63 | 5.18 | 2.88 | 2.42 | 3.24 | 11.22 | 9.48 | 12.90 |
| 10 - 29 | *N* | | 196 | |  |  | 194 |  |  | 196 |  |  | 194 |  |  |
|  | Mean | | 29.82 | | 28.95 | 30.64 | 28.74 | 28.20 | 29.26 | 18.83 | 18.35 | 19.26 | 77.28 | 75.97 | 78.71 |
|  | SD | | 5.83 | | 5.30 | 6.33 | 3.78 | 3.40 | 4.17 | 3.15 | 2.88 | 3.40 | 10.05 | 8.97 | 11.14 |
| 30 + | *N* | | 55 | |  |  | 55 |  |  | 55 |  |  | 44 |  |  |
|  | Mean | | 31.33 | | 29.61 | 33.14 | 30.18 | 29.39 | 30.93 | 17.98 | 17.08 | 18.81 | 79.49 | 77.43 | 81.63 |
|  | SD | |  | |  |  | 3.29 | 2.79 | 3.69 | 3.23 | 2.69 | 3.70 | 8.49 | 7.10 | 9.53 |
| Total | *N* | | 299 | |  |  | 297 |  |  | 299 |  |  | 297 |  |  |
|  | Mean | | 30.13 | | 29.49 | 30.85 | 28.69 | 28.25 | 29.10 | 18.98 | 18.60 | 19.35 | 77.73 | 76.74 | 78.66 |
|  | SD | | 5.93 | | 5.49 | 6.37 | 3.93 | 3.61 | 4.24 | 3.22 | 2.99 | 3.46 | 9.98 | 9.14 | 10.87 |

## Supplementary Table 15. Time since first public performance: ANOVA Post-hoc tests with Bonferroni correction and CI through Bootstrapping

|  |  | Personal Resources | | | | External Resources | | | |  |
| --- | --- | --- | --- | --- | --- | --- | --- | --- | --- | --- |
|  |  |  | | BCa 95% Confidence Interval | |  | | BCa 95% Confidence Interval | |  |
| (I) Years since first concert | (J) Years since first concert | Mean Dif.  (I-J) | *p* | Lower | Upper | Mean Dif.  (I-J) | *p* | Lower | Upper | |
| 1 - 9 years | 10 - 29 years | -1.992* | .004 | -3.516 | -.545 | 1.903* | <.001 | .932 | 2.816 | |
|  | 30 + | -3.432* | <.001 | -5.080 | -1.846 | 2.747* | <.001 | 1.574 | 3.987 | |
| 10 - 29 years | 1 - 9 years | 1.992* | .004 | .699 | 3.271 | -1.903* | <.001 | -2.810 | -.942 | |
|  | 30+ | -1.440* | .042 | -2.451 | -.376 | .845 | .233 | -.060 | 1.782 | |
| 30 + | 1 - 9 years | 3.432* | <.001 | 1.996 | 4.870 | -2.474* | <.001 | -3.978 | -1.584 | |
|  | 10 - 29 years | 1.440 | .042 | .502 | 2.364 | -.845 | .233 | -1.785 | .069 | |

## Supplementary Table 16. Practice Hours per day X Expertise: chi-square

|  |  | Expertise | | |
| --- | --- | --- | --- | --- |
| Practice hours per day |  | Students | Pre-Professional | Professional |
| Less than 1 hour |  | 5 | 10 | 33 |
|  |  |  |  |  |
|  |  | -1.7 | -1.0 | 2.2 |
| 1 - 2 hours |  | 9 | 26 | 48 |
|  |  | -2.3 | 1.1 | .8 |
|  |  |  |  |  |
| 2 - 3 hours |  | 18 | 24 | 38 |
|  |  | .8 | .8 | -1.4 |
|  |  |  |  |  |
| 3 - 4 hours |  | 18 | 15 | 26 |
|  |  | 2.4 | -.2 | -1.7 |
|  |  |  |  |  |
| More than 4 hours |  | 8 | 5 | 17 |
|  |  | 1.1 | -1.3 | .3 |
|  |  |  |  |  |

## Supplementary Table 17. Mean for scores in all factors.

|  |  | Practice Organization | Personal Resources | External Resources | Total |
| --- | --- | --- | --- | --- | --- |
| N | Valid | 299 | 297 | 299 | 297 |
|  | Missing | 1 | 3 | 1 | 3 |
| Mean |  | 30.13 | 28.69 | 18.98 | 77.73 |
| SD |  | 5.93 | 3.93 | 3.22 | 9.98 |
| Minimum |  | 10 | 14 | 10 | 40 |
| Maximum |  | 40 | 35 | 25 | 100 |
